# Supramolecular Chirality Transfer toward Chiral Aggregation: Asymmetric Hierarchical Self‐Assembly

**DOI:** 10.1002/advs.202002132

**Published:** 2021-03-01

**Authors:** Shuai Huang, Haifeng Yu, Quan Li

**Affiliations:** ^1^ School of Materials Science and Engineering Key Laboratory of Polymer Chemistry and Physics of Ministry of Education Peking University Beijing 100871 China; ^2^ Institute of Advanced Materials School of Chemistry and Chemical Engineering Southeast University Nanjing Jiangsu Province 211189 China; ^3^ Advanced Materials and Liquid Crystal Institute and Chemical Physics Interdisciplinary Program Kent State University Kent OH 44242 USA

**Keywords:** asymmetric spatial structure, chiral aggregation, chirality transfer, hierarchical self‐assembly

## Abstract

Self‐assembly, as a typical bottom‐up strategy for the fabrication of functional materials, has been applied to fabricate chiral materials with subtle chiral nanostructures. The chiral nanostructures exhibit great potential in asymmetric catalysis, chiral sensing, chiral electronics, photonics, and even the realization of several biological functions. According to existing studies, the supramolecular chirality transfer process combined with hierarchical self‐assembly plays a vital role in the fabrication of multiscale chiral structures. This progress report focuses on the hierarchical self‐assembly of chiral or achiral molecules that aggregate with asymmetric spatial structures such as twisted bands, helices, and superhelices in different environments. Herein, recent studies on the chirality transfer induced self‐assembly based on a variety of supramolecular interactions are summarized. In addition, the influence of different environments and the states of systems including solutions, condensed states, gel systems, interfaces on the asymmetric hierarchical self‐assembly, and the expression of chirality are explored. Moreover, both the driving forces that facilitate chiral bias and the supramolecular interactions that play an important role in the expression, transfer, and amplification of the chiral sense are correspondingly discussed.

## Graphical abstract

Supramolecular and aggregation chirality, that emerges from an initial chiral bias, leads to a variety of hierarchically self‐assembled structures through a supramolecular chirality transfer, which is significantly influenced by different environments. For example, chiral assemblies are favored in solution, helical structures in the condensed state, chiral networks in gel, and chiral arrangement at the interface.

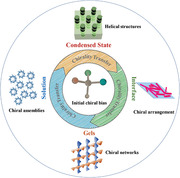

## 1 Introduction

Chirality is an inherent characteristic of biological systems, which has been expressed at every structural level in nature, including molecules, supramolecules, microscopic assemblies, macroscopic matter, and even galaxies, with sizes ranging from angstroms to light years. These chiral structures demonstrate a close relationship with the origin and the evolution of life, which has intrigued persons for numerous years. Generally speaking, chirality can be generated by breaking the *S_n_* symmetry. In most cases, an object is considered chiral if it loses both the mirror plane (*σ* or *S*_1_) and inversion (i or *S*_2_) symmetries.^[^ ^1^ ^]^ Consequently, the lack of symmetry elements results in the formation of chirality through multiple length scales. Typical chiral structures in biology such as the *α*‐helix of peptides, double helix of DNA, and triple helix of collagens are predominantly at the nanoscale level. Further exploration of artificial materials with elaborate chiral nanostructures in nanoscale has revealed their outstanding functions that can be applied in, inter alia, asymmetric catalysis,^[^ ^2^ ^]^ chiral sensing,^[^ ^3^ ^]^ chiral electronics,^[^ ^4^ ^]^ and photonics.^[^ ^5^ ^]^ Hence, the assembly of asymmetric nanostructures has been becoming a critical subject in the fabrication of chiral functional devices.

**Figure 1:**
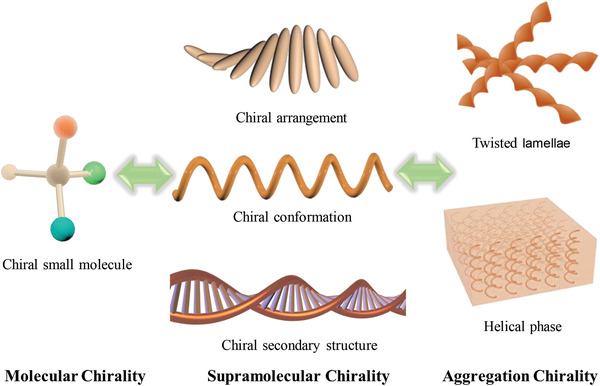
Schematic illustration of chirality from molecular to supramolecular and aggregation levels. From left to right: molecular chirality resulting from chiral center or chiral axis; supramolecular chirality including the chiral arrangement of molecules, the chiral main chain formation of macromolecules and their secondary structures; and the aggregation chirality of some twisted structures and helical structures, respectively.

Top‐down and bottom‐up approaches are currently the most accepted strategies for the fabrication of hierarchical micro‐structured materials. However, nanoscale structures are too small to be easily modified by the typical top‐down method. However, self‐assembly has demonstrated its superiority in this field as one of the most elegant bottom‐up methods. For example, amphiphilic molecules, liquid crystals, and block copolymers (BCs) can self‐segregate into ordered nanostructures, both in solutions and condensed states, including micelles, vesicles, close‐packed spheres, cylinders, gyroids, and lamellae.^[^ ^6^ ^]^ However, one of the most favorable ways to introduce chirality into these nanoarchitectures is by structural symmetry breaking. Molecular chirality can be achieved by introducing a chiral center or axis into molecules using an appropriate synthesis strategy.^[^ ^7^ ^]^ However, noncovalent interactions such as hydrogen bonding, coordination interactions, electrostatic interactions, and van der Waals forces can assist in transferring the introduced chirality to the achiral molecules in supramolecular systems, which is termed the induced supramolecular chirality. In addition, the symmetry of the assemblies of achiral molecules are susceptible to breakage by environmental effects like solvents, mechanical forces, and circularly polarized light (CPL), referred to as the induced aggregation chirality. These three types of chirality are not independent of each other, but are closely correlated because of the ability of these systems to self‐assemble into chiral aggregates through a hierarchical chirality transfer process. Therefore, throughout this progress report, we make use of the terms molecular chirality, supramolecular chirality, and aggregation chirality to distinguish between the length scales where chirality information is expressed, as shown in **Figure** **1**.

During the fabrication of chiral nanostructures, chirality transfer and amplification are expected to facilitate the expression of asymmetry at aggregation levels. It has been validated that these chirality transfer and amplification phenomena from molecular to aggregation level should obey two principles, that is., the “sergeant and soldiers” effect^[^ ^8^ ^]^ and the majority rule principle.^[^ ^9^ ^]^ These principles were first established by Green et al. during their study on dynamic helical polyisocyanate systems. The original “sergeant and soldiers” effect describes that the introduction of a few chiral units (the sergeants) can induce a large number of achiral units (the soldiers) in a dynamic helical chain to adopt a preferred‐handed chiral conformation. The concept has subsequently been extended to all supramolecular systems, which only require a small number of chiral molecules (the sergeants) to create the chiral bias and induce the preferred handedness of the supramolecular structure of achiral building blocks (the soldiers).^[^ ^10^ ^]^ For example, one type of disc‐shaped chiral molecule could form helical columnar stacks in nonpolar solvents and generate a strong Cotton effect in circular dichroism (CD) spectra, whereas its achiral isomer could only form CD‐silent racemic structures. However, a preferred one‐handed helical aggregate with an intense CD signal was observed when 2.5% of the chiral isomers were added to the achiral isomers, demonstrating the “sergeant and soldiers” effect, as reported by Meijer et al.^[^ ^10d^ ^]^ The majority rule principle implies that the handedness of the majority enantiomers dominate the helicity of the minority chiral units in the dynamic helical chain, which is also applicable in supramolecular chiral systems.^[^ ^11^ ^]^ In majority rule experiments, for instance, the enantiomers were mixed with varying molar ratios, where the anisotropy factor (g), determined as a function of the enantiomeric excess value (ee) of the mixtures, showed a nonlinear dependence on the ee.^[^ ^11d^ ^]^ Over the past several decades, the chirality transfer and amplification phenomena based on the “sergeant and soldiers” effect and the majority rule principle have been extensively studied in a diverse variety of self‐assembled systems based on covalent bonding and noncovalent supramolecular interactions.

Through noncovalent supramolecular interactions like hydrogen bonding, *π*—*π* stacking, and solvation, molecules first amass to form supramolecular clusters, which may then function as building blocks for further assembly into aggregate structures at the nanoscale level. When the chirality is transferred and amplified, the chirality of the building blocks typically determines that of the nano‐aggregates such as the rotational direction of a helix. However, at times, the aggregation chirality is significantly influenced by the ambient environment or external stimuli, which results in symmetry breakage in aggregates assembled by achiral molecules, thus leading to the chirality reversal behavior of some chiral systems.^[^ ^12^ ^]^

Since the design strategy for various types of chiral building blocks and recent progresses in their self‐assembly have been reviewed by Liu^[^ ^10b,c^ ^]^ and Sánchez,^[^ ^12d,e^ ^]^ we will not concentrate on the aforementioned. This progress report will focus on the chirality transfer behaviors of chiral systems under different ambient environments due to the extraordinary effects of the aforementioned environments on the asymmetric hierarchical self‐assembly. Herein, we will describe recent advances in the asymmetric hierarchical self‐assembly induced by supramolecular chirality transfer in solutions, condensed states, gels, and at interfaces, in this order. In addition, we will discuss how the chirality information gets transferred into aggregate structures. Stimulus‐responsive properties of these chiral nanostructures will be also summarized accordingly.

## 2 Supramolecular Chirality Transfer in Solution Systems

Herein, the solution systems refer to homogeneous solutions of solutes molecularly dispersed in solvents as well as, inter alia, micro‐assemblies such as micelles, vesicles, and nanoparticles, which are dispersed in solvents as nanoscale aggregates. Chirality transferring behaviors in noncovalent bonding, *π*—*π* stacking, and the entanglement of hydrophobic molecular chains have recently attracted significant attention because supramolecular self‐assembly is mostly investigated in diverse solvents driven by the aforementioned interactions.^[^ ^13^ ^]^ According to previous research, there are numerous factors that have a prominent effect on the self‐assembly and supramolecular chirality transfer process, including the solubility, polarity, and chirality of the solvent, the supramolecular interactions, and the interfacial interaction between the aggregates and the solvent.^[^ ^10^, ^12^, ^13^ ^]^ Therefore, several studies have been conducted to investigate the influence of these factors, thereby enabling further comprehension of the assembly behaviors in solution systems induced by the chirality transfer.

### 2.1 Assembly of Chiral Molecules in Achiral Solvents

Since all aggregation types can be driven by various supramolecular interactions in solvent environments, the inherent chirality of compounds often aids in transferring the asymmetric information from molecular to aggregate level, thus leading to different morphologies. These processes are significantly influenced by the solvent effect. Self‐assembled supramolecular structures are often multifarious due to the difference in compatibility between solutes and solvents. In chiral systems, chirality transfer can be simultaneously impacted. Recently, Meijer et al. reported a supramolecular polymer system with chiral‐amide functionalized zinc porphyrin derivatives as the monomer, which exhibited strong switch‐like behavior through competing aggregation pathways in different solvents.^[^ ^14^ ^]^ At low temperatures under poor solvent conditions, (*S*)‐monomers assemble into H‐aggregate nucleated polymers, thus forming hierarchical superhelices. In contrast, weakly coupled J‐type aggregates formed through an isodesmic aggregation process with intermediate solvent quality and temperatures, without the expression of supramolecular chirality. These results demonstrate the significant effect of different assembly routes from molecules to aggregates on the hierarchical chirality transfer process, which may contribute to improved understanding of the interference between self‐assembly and chirality transfer process.

**Figure 2:**
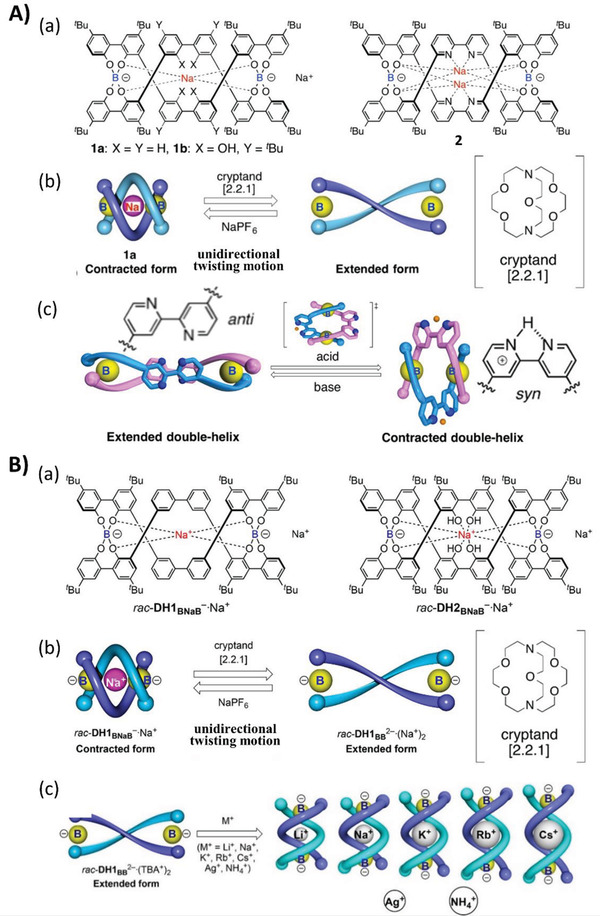
A) (a) Chemical structures of double‐stranded spiroborate helicates. (b) Schematic representation of the unidirectional spring‐like motion upon Na^+^‐ion release and binding. (c) Schematic representation of acid/base‐triggered reversible extension‐contraction motion. Adapted with permission.^[^ ^16^ ^]^ Copyright 2016, American Chemical Society. B) (a) Chemical structures of double‐stranded spiroborate helicates *rac*‐DH1_BNaB_^−^·Na^+^ and *rac*‐DH2_BNaB_^−^·Na^+^. (b) Schematic representation of the unidirectional spring‐like motion upon Na^+^‐ion release and binding. (c) Schematic representation of the inclusion complex formation of *rac*‐DH1_BB_^2−^(TBA^+^)_2_ with alkali metals, Ag^+^, and NH_4_^+^ ions. TBA: tetrabutylammonium. Adapted with permission.^[^ ^17^ ^]^ Copyright 2018, American Chemical Society.

Zhang et al. also confirmed that the solubility of solvents and the dispersed state of chiral molecules in solvents could influence the expression of hierarchical chirality. They observed that chiral polyfluorene derivatives were optically inactive in molecularly dispersed solutions, but exhibited intense CD signals after aggregation or coating with selected solvents. However, the difference in solubility could even induce conformation‐driven mirror‐image supramolecular chirality isomerism by confining the intrinsically chiral macromolecules in a metastable *β*‐phase.^[^ ^15^ ^]^ In addition to solvent properties, other factors like pH and metal ions could alter the environment of the solution systems, further exerting profound impacts on the transfer and expression of chirality. Yashima et al. reported the extension and contraction motions of the spiroborate‐based double‐stranded helicates composed of 4,4′‐linked 2,2′‐bipyridine and its *N,N′*‐dioxide units in the middle of ortho‐linked tetraphenol strands, which are regulatable by the binding and releasing of protons or metal ions, as shown in **Figure** **2**A.^[^ ^16^ ^]^ The ion species demonstrate a remarkable dependence on the ion‐assisted extension‐contraction motions in these spring‐like systems, as shown in Figure 2B.^[^ ^17^ ^]^ These phenomena arise from the occurrence of anti‐syn conformational changes at the linkages, which are amplified into a large‐scale molecular motion of the helicates, thus leading to reversible spring‐like motions. These motions suggest that ionic conditions remarkably influence the asymmetric supramolecular structures in solution through dynamic supramolecular interactions, and might provide a coherent strategy for developing functional chiral materials with controllable supramolecular helical structures.

The control of helical conformation can be achieved by tuning the intermolecular and intramolecular interactions between the solvent and solute molecules. Wan et al. synthesized a pair of enantiomeric *cis*‐poly(phenylacetylene)s (PPAs) substituted at the meta positions of pendant phenyl rings by an achiral methoxycarbonyl group and a chiral 1‐methylpropyloxycarbonyl group (i.e., sP‐Me‐C4/rP‐Me‐C4). They also synthesized two *cis*‐PPAs bearing either a methoxycarbonyl (i.e., *m*‐aP‐Me) or a 1‐methylpropyloxycarbonyl (i.e., *m*‐sP‐C4) meta substituent. Wan et al. observed that sP‐Me‐C4/rP‐Me‐C4 tended to adopt contracted cis‐cisoid helical conformations in hydrogen‐bond accepting solvents or weak hydrogen‐bond donating solvents where the intramolecular n→π* interactions were favored. However, sP‐Me‐C4/rP‐Me‐C4 adopted *cis*‐transoid helical conformations in hydrogen‐bonding donating solvents where the n→π* interactions were disrupted by the strong hydrogen bonding between the solvent and solute molecules.^[^ ^18^ ^]^ Furthermore, temperature also had a significant impact on the assembled helical structures in this system.

The introduction of chiral additives to achiral polymers, with the aid of strong supramolecular interactions, is able to induce transfer and amplification in solution systems. Wan et al. reported the asymmetric induction of supramolecular chirality through ionic interactions between pyridinium pendants in isotactic poly(2‐vinylpyridine) and chiral acid ions from (+)‐camphorsulfonic acid and dodecylbenzensulfonic acid. These interactions forced the polymer backbone to twist in a preferred handedness manner in CHCl_3_ and the mixed solvent CHCl_3_/CH_3_CN.^[^ ^19^ ^]^ It was demonstrated that many factors including the base to acid ratio, chiral‐acid content, and solvent nature could influence the induced chirality. In addition, by tuning the solvent composition, four distinct types of “sergeants‐and‐soldiers” effect were observed within a single system for the first time, due to the sensitivity of ionic interactions to solvent polarity. Importantly, the typical isotactic poly(2‐vinylpyridine) plastic was successfully transformed into functional polymer materials, thus helping to extend the design strategy for achieving chiroptical polymers with helical supramolecular structures.

The chiral structures fabricated in solutions are usually based on weak noncovalent bonding interactions, which are not stable enough for practical applications. Therefore, Yashima et al. proposed a solution method for the fabrication of stabilized chiral organic nanotube systems. They reported a simple “helix‐to‐tube” strategy to construct covalent organic nanotubes from poly(*m*‐phenylene diethynylene)s (poly‐PDEs) containing chiral amide side chains, which typically adopted a helical conformation through hydrogen‐bonding and solvophobic interactions in specific solvents. By photo‐crosslinking the 1,3‐butadiyne moieties aligned longitude across the entire helix with actinic light, the helically‐folded polymer chains formed inherently stable chiral covalent nanotubes.^[^ ^20^ ^]^

### 2.2 Chiral Solvent‐Induced Chirality Transfer

**Figure 3:**
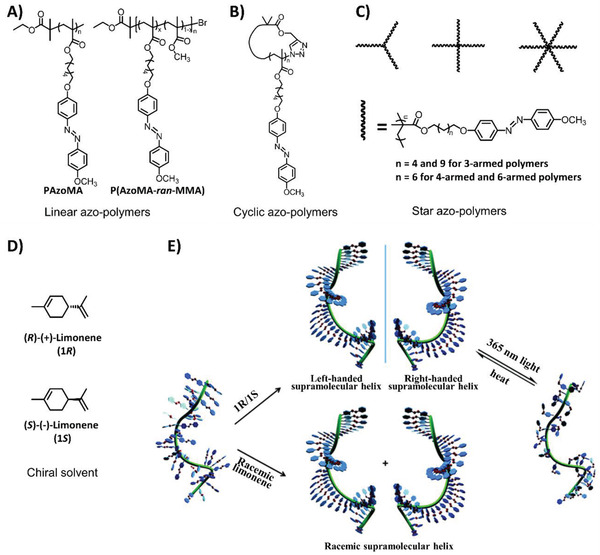
A) Chemical structures of achiral linear azo‐polymers. Reproduced with permission.^[^ ^21^ ^]^ Copyright 2015, Royal Society of Chemistry. B) Chemical structures of cyclic azo‐polymers. Reproduced with permission.^[^ ^22b^ ^]^ Copyright 2018, Royal Society of Chemistry. C) Chemical structures of achiral star azo‐polymers. Reproduced with permission.^[^ ^22a^ ^]^ Copyright 2015, Royal Society of Chemistry. D) Chemical structures of the chiral solvent limonene. E) Schematic illustration of the supramolecular helical structures of the inherently achiral linear azo‐polymer PAzoMA induced by chiral limonene, and the chiroptical switch behaviors. The polymethacrylate main chain is not involved in the helical organization. Adapted with permission.^[^ ^21^ ^]^ Copyright 2015, Royal Society of Chemistry.

Generally, achiral molecules rarely form supramolecular assemblies and aggregates with hierarchical chirality. However, the symmetry of achiral supramolecular assembles is susceptible to breakage in asymmetric environments, particularly in solution systems, where the surroundings are conveniently adjusted by the regulation of the property of solutes and solvents. As a result, the utility of chiral solvents is an effective strategy for creating an asymmetric environment. Zhang et al. investigated the chirality transfer from chiral solvent molecules (limonene) to achiral side‐chain azobenzene‐containing polymers (**Figure** **3**).^[^ ^21^ ^]^ They observed that the preferred supramolecular chirality of the assemblies of trans‐azobenzene units was generated and determined by the limonene chirality, as shown in Figure 3A,E. Further studies on cyclic (Figure 3B) and star‐shaped polymers (Figure 3C) containing azobenzene moieties in similar chiral solvents and cosolvents demonstrated that chirality transfer and amplification from chiral solvent molecules was not limited to linear polymers.^[^ ^22^ ^]^ Hence, this provided a universal method to fabricating hierarchical chiral aggregation from polymers with a variety of topological chain structures. Nevertheless, other structural factors including the spacer lengths and push–pull electronic substituents were confirmed to impact the chirality transfer and aggregation process.^[^ ^23^ ^]^ Short spacer lengths or appropriate terminal groups like CH_3_ or H groups restricted the assembly of azobenzene units and hindered supramolecular chirality transfer. Hence, the appropriate spacer lengths and push–pull electronic substituents could be manipulated to control the induction of supramolecular chirality by chiral solvation. Furthermore, the photoisomerization of azobenzene moieties endow these systems with photoresponsive properties, thus facilitating the fabrication of photo‐regulatable chiral supramolecular assemblies. These reports all contributed to perfecting the principles for designing and constructing functional chiroptical materials from achiral polymers.

### 2.3 Chiral Assemblies Triggered by External Stimuli

As reported decades ago, the symmetry breaking of hierarchical structures assembled from achiral or racemic molecules in achiral solvents could be induced by external stimuli such as hydrodynamic flow by mechanical stirring,^[^ ^12^, ^24^ ^]^ photoirradiation using CPL,^[^ ^25^ ^]^ and magnetic field.^[^ ^26^ ^]^ Hence, a diversity of physical fields has been applied to generate and amplify chiral bias with carefully investigated processes and mechanisms. For instance, photoirradiation is one of the most effective and clean methods, which is also closely related to the origin of life. Thus, photoinduced chiral assembly in solution systems has attracted significant interest in recent years.

**Figure 4:**
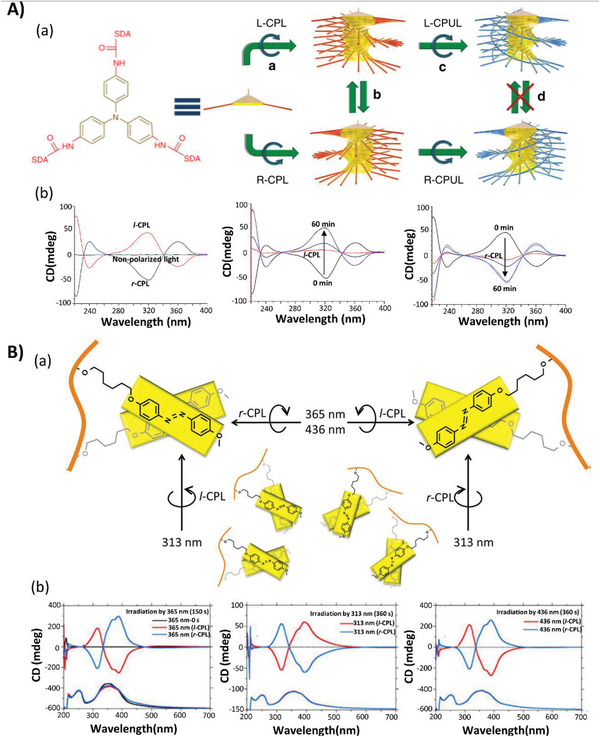
A) Manipulation of the supramolecular chirality of the assemblies of tris(4‐trideca‐4,6‐diynamidophenyl)amine by light. (a) Schematic illustration of induction, control, and locking of supramolecular chirality by circularly polarized light. In this figure, CPL refers to circularly polarized light and CPUL refers to circularly polarized ultraviolet light. (b) CD spectra after irradiation with non‐polarized light (black), *l*‐circularly polarized light (red), and *r*‐circularly polarized light (blue) for 10 min in 1,2‐dichloroethane. Adapted with permission.^[^ ^25c^ ^]^ Copyright 2015, Springer Nature. B) The induced bisignate Cotton effect of an achiral polymethacrylate carrying achiral azobenzene side chains. (a) Schematic illustration of the induced aggregation chirality by circularly polarized light at different wavelengths (313, 365, 405, and 436 nm). (b) Corresponding CD and UV–vis spectra after being irradiated by *l*‐ and *r*‐circularly polarized light at different wavelengths. Adapted with permission.^[^ ^12a^ ^]^ Copyright 2017, American Chemical Society.

The supramolecular chirality of polymer systems can be generated by the photoirradiation of CPL. Kim et al. designed a system composed of one *π*‐conjugated molecule tris(4‐trideca‐4,6‐diynamidophenyl)amine, where the entire process of induction, control, and the locking of supramolecular chirality could be manipulated by actinic light.^[^ ^25c^ ^]^ Upon the irradiation of circularly polarized visible light, the supramolecular chirality of the resulting aggregates could be selectively and reversibly controlled by its rotational direction. However, the supramolecular chirality could be locked by irradiation with circularly polarized ultraviolet (UV) light, as shown in **Figure** **4**A. In this system, the chiral bias generated by CPL could not only be transferred to a supramolecular level, but also considerably amplified when combined with a self‐assembly process.

Recently, Fujiki et al. reported an enhanced bisignate Cotton effect of an achiral polymethacrylate carrying achiral azobenzene side chains with restricted conformational freedom in aggregates irradiated by right or left CPL at four wavelengths (313, 365, 405, and 436 nm).^[^ ^12^ ^]^ In addition, they systematically studied the influence of irradiation wavelength and exposure time, as shown in Figure 4B.^[^ ^12a^ ^]^ They demonstrated that a chiral azobenzene with a high dissymmetry ratio could be generated, inverted, and switched in multiple cycles by photoirradiation with weak monochromatic incoherent CPL over a short period. Herein, they demonstrate the advantages of using CPL sources with multiple wavelengths rather than a single wavelength in achieving elaborate chiroptical functions including chiroptical polarization, restoration, inversion, switching, and long‐term memory in such azobenzene‐containing aggregates.

Moreover, the photophysical control of all chiroptical polarization, depolarization, inversion, retention and switching in the ground or the photoexcited states of assemblies in solutions and aggregates has also been achieved. Fujiki et al. reported the photo‐manipulation of chiral assembly in the microparticles of CPL‐dependent chiroptical poly[(9,9‐di‐*n*‐octylfluoren‐2,7‐diyl)‐alt‐bithiophene] (PF8T2) dispersed in optofluidically tuned CHCl_3_‐MeOH cosolvents.^[^ ^27^ ^]^ They indicated that wavelength selection and the handedness of CPL may lead to the inversion of chirality. In addition, both left‐ and right‐handed CPL photons could efficiently induce responsive behaviors including chiroptical polarization, depolarization, inversion, long‐term retention, and switching modes into achiral PF8T2 microparticles. Moreover, suitable cosolvents might enhance the induced CD signals due to the optofluidic effect.

## 3 Hierarchical Chirality Transfer in Condensed States

In contrast to solution systems, the materials in condensed states are devoid of solvents, which restricts the potential nanostructures that can be fabricated. Therefore, annealing methods including thermal and solvent annealing are the most effective approaches to providing sufficient free volume for accelerating the supramolecular self‐assembly. When incorporated with the introduction of chiral bias, self‐assembly processes could proceed, accompanied by hierarchical chirality transfer through various supramolecular interactions.^[^ ^28^ ^]^ However, a unique design strategy is often required for accomplishing the chirality induced assembly, considering the restriction of molecular motion in condensed states. In this section, we will summarize recent progresses in the self‐assembly of small molecules and polymers in the condensed state along with the participation of chiral sense.

### 3.1 Chiral Assemblies from Molecular Chirality in Condensed States

In condensed states, an asymmetry in the special shape can be amplified into supramolecular chirality, resulting in the formation of chiral bias. Shen et al. studied the shape effect of a large molecule on the self‐assembly of supramolecules using a bent‐core molecule, which was covalently linked to a disc‐like hexa‐*peri*‐hexabenzocoronene, thus yielding a disc‐bent core amphiphile.^[^ ^29^ ^]^ It was confirmed that this molecular morphology could induce hierarchical chirality and lead to the formation of a columnar phase, which exhibited a 2D nano‐ribbon with spiral texture resulting from the double‐stranded supracolumns.

Supramolecular chirality transfer in polymer systems has significant potential for detecting special handedness, which is almost impossible using conventional spectroscopic methods. Maeda et al. reported the first direct chirality sensing of a series of chiral hydrocarbons and isotopically chiral compounds (deuterated versions of parent compounds) by a stereoregular polyacetylene bearing 2,2′‐biphenol‐derived pendants.^[^ ^30^ ^]^ The aforementioned compounds were termed “hidden chirality” because of the difficulty to be detected.^[^ ^30^ ^]^ The chirality detection using polyacetylene was based on the generation of an excess one‐handed helix induced by the chiral hydrocarbons and deuterated isotopomers by the transfer and amplification of chirality, followed by its static memory of the induced chirality. As a result, chiral information and the hidden chirality could be stored as an excess of a single‐handed helix memory in the system for an extended period.

CPL can also be used to generate supramolecular and aggregation chirality in condensed states assisted by the introduction of photoresponsive liquid‐crystalline units, based on the fluidity of the liquid crystal phase and the unique photo‐orientation property of mesogens. Pinol et al. synthesized a series of liquid‐crystalline polymers with a repeating group consisting of two functional units, with at least one of them being an azobenzene moiety.^[^ ^31^ ^]^ The photoinduced chiral signals were generated upon the photoirradiation of CPL at room temperature.^[^ ^32^ ^]^ However, the induced chirality did not demonstrate excellent stability because of the relaxation of segments in liquid‐crystalline homopolymers. BCs could help to limit relaxation by confining the photo‐addressable segments into nanoscale microphase separation, whereas alternative copolymers containing photo‐crosslinkable cinnamate moieties could help to fix the induced chirality. As a result, BCs and alternative copolymers were synthesized to facilitate an excellent stability of the photoinduced supramolecular chirality in these liquid‐crystalline polymers.

### 3.2 Crystallization with the Participation of Chirality

**Figure 5:**
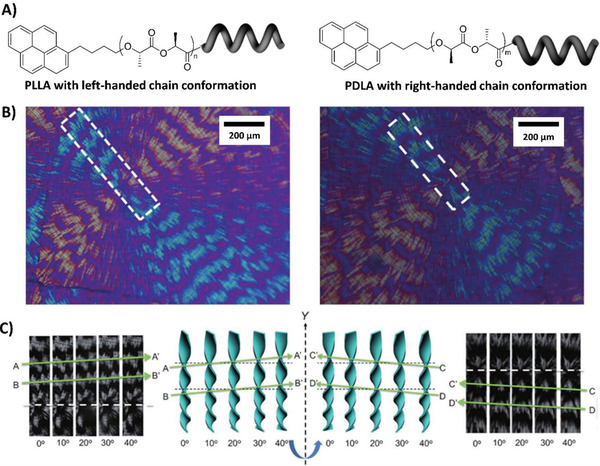
A) Chemical structures of pyrene‐labeled PLLA and pyrene‐labeled PDLA. B) POM images of PLLA (left) and PDLA (right) isothermally crystallized at 110 °C. The delimited areas represent the observed slices during the rotation experiment. C) Vertical sections of PLLA (left) and PDLA (right) spherulites observed by POM during the rotation. The value at the bottom represents the angle of twist around the Y axis in the right‐handed positive sense. Adapted with permission.^[^ ^32^ ^]^ Copyright 2014, Wiley‐VCH.

Poly‐*l*‐lactic acid (PLLA) and poly‐*d*‐lactic acid (PDLA) are a typical category of chiral polymers with favorable crystallization properties. Chiral bias in the main chain that exerts a profound effect on the crystallization process of PLLA or PDLA. Ho et al. systematically investigated the asymmetrical crystallization of polylactides. With the assistance of a polarized optical microscope equipped with a home‐made goniometer, the formation of left‐ and right‐handed helical superstructures (twisting lamellae) in the banded spherulites of the pyrene‐labeled PLLA and PDLA are directly observed, as shown in **Figure** **5**.^[^ ^32^ ^]^ The occurrence of twisting and shifting with preferential direction was ascribed to the imbalanced stress at the opposite folding surface along with J‐type aggregation. Molecular chirality evolved into hierarchical aggregation chirality during the self‐assembly process of enantiomeric polylactides (PLA) after being directed by the twisting and shifting mechanism. Moreover, the banded spherulites resulting from lamellar twisting due to imbalanced stresses at the opposite folding surface were also observed in the co‐crystalline enantiomeric PLA blended with poly(ethylene glycol), indicating a chirality transfer process based on intermolecular chiral interactions during isothermal crystallization.^[^ ^33^ ^]^

Through supramolecular interactions, chirality can be transferred to an achiral polymer chain and the corresponding polymer crystals. For example, when (*R*)‐ or (*S*)‐hexahydromandelic acid (HMA), which can associate with isotactic poly(2‐vinylpyridine) (*i*P2VP), was introduced as a chiral dopant, mirror‐image CD spectra in the complex systems were observed.^[^ ^34^ ^]^ Moreover, a preferential sense of the twisted crystalline lamellae was also obtained in the *i*P2VP/HMA complex, thus suggesting the homochirality evolution from conformational to hierarchical chirality. The handedness of twisted lamellae in banded spherulites could be controlled by the chiral dopants.

The asymmetric crystallization process can assist the selective chiral separation of optically pure enantiomers. Wan et al. proposed a self‐reporting strategy for collecting two optically pure enantiomers in a single crystallization process by visualizing the crystallization process with the assistance of a dyed self‐assembled inhibitor.^[^ ^35^ ^]^ This inhibitor was synthesized from copolymers with tri(ethylene glycol)‐grafting polymethylsiloxane and poly(*N*‐6‐methacryloyl‐*L*‐lysine) as the main and side chains, respectively.^[^ ^35^ ^]^ The inhibitor could induce a complete isolation of the two enantiomers through the selective fractional crystallization of racemic aggregates, and could also selectively label the latterly precipitated product so that the crystals of the second enantiomer yielded a direct color readout. Moreover, Wan et al. further developed a quantitative chiral separation method by the enantiomer‐selective magnetization of racemic aggregates.^[^ ^36^ ^]^ The assembled magnetic nano‐splitters consisted of magnetic nanoparticles, hydrophobic Fe_3_O_4_@oleic acid, as the core and the polymeric inhibitors, and amphiphilic poly(*N*‐6‐methacryloyl‐*S*‐lysine)‐block‐polystyrene, with high stereoselectivity for the target crystals, as the shell. The nano‐splitters resulted in distinct crystallization processes for the two enantiomers under selective magnetic fields. Thus, the simple and quantitative separation of the *R*‐ and *S*‐crystals in one single crystallization process was successfully accomplished. Consequently, these findings provide novel insights into the design and development of functional materials for multiscale chiral resolution based on hierarchically self‐assembled nanoarchitectures.

### 3.3 Asymmetric Phase Structures

Well‐defined BCs can usually self‐assemble into various ordered nanostructures, demonstrating significant potential as nanotemplates for the fabrication of ordered nanomaterials with desirable patterning and arrays. The introduction of chiral bias might result in an asymmetric hierarchical self‐assembly process, thereby enriching the structures that can be formed by BCs in condensed states. Ho et al. conducted one of the first studies on the phase structures of chiral BCs and achieved the fabrication of helical phases in the BCs of PLLA block polystyrene (PLLA‐*b*‐PS) or PDLA block polystyrene (PDLA‐*b*‐PS).^[^ ^37^ ^]^ However, there are numerous factors that can impact the helical phase in these chiral BCs, such as the volume ratio of the two blocks. Recently, Ho et al. investigated the competitive interactions of *π*—*π* junctions between the chiral PLA and achiral PS blocks, and their roles on microphase‐separation behaviors.^[^ ^38^ ^]^ They indicated that the formation of *π*—*π* stacking based on *π*—*π* junctions would reduce the structural ordering due to the mixing of constituent blocks. However, ordered morphologies were easily obtained in BCs containing alkyl junctions where no *π*—*π* stacking could occur, thus indicating the competitive interactions of *π*—*π* junctions and the microphase separation of chiral BCs. The interface between chiral PLA and achiral PS domains were then investigated by designing a series of PLA‐containing BCs with various sequences and molecular weights of chiral PLLA and achiral poly(*d*, *l*‐lactide) (PDLLA) blocks. They observed that the direct connection of a chiral block to a PS block in the order PS−PLLA−PDLLA produced a chiral interface, thereby enabling a short PLLA block to induce the helical phase, due to the significant effect of helical steric hindrance on self‐assembly. In contrast, the self‐assembled morphology of the chiral BC in the sequence PS−PDLLA−PLLA was determined by the length of the PDLLA block. Consequently, only a long PLLA block was able to induce the helical phase due to the absence of a chiral interface.^[^ ^39^ ^]^

Another important factor in chiral BCs that impacts the phase structures is the crystallization of the chiral block. It was reported that the helical phase in chiral PLA‐based BCs was just a long‐lived intermediate state.^[^ ^40^ ^]^ Ho et al. recently synthesized poly(cyclohexylglycolide) (PCG)‐based chiral BCs, poly(benzyl methacrylate)‐*b*‐poly(d‐cyclohexylglycolide) (PBnMA‐*b*‐PDCG), and PBnMA‐*b*‐poly(l‐cyclohexyl glycolide) (PBnMA‐*b*‐PLCG) for comparison with chiral PLA‐based BCs. Furthermore, they studied the homochirality evolution from monomeric, to conformational, and to meso‐domain chirality across multiple size scales during self‐assembly processes.^[^ ^41^ ^]^ By eliminating the crystallization of the chiral block, they observed the formation of an equilibrium helical phase. In PBnMA‐*b*‐PLCG and PBnMA‐*b*‐PDCG, the bulkier chiral side group of the PCG might generate a more persistent helical bias, which could assist in strengthening the chiral anisotropy of intersegment forces, thereby leading to chiral meso‐domains.

**Figure 6:**
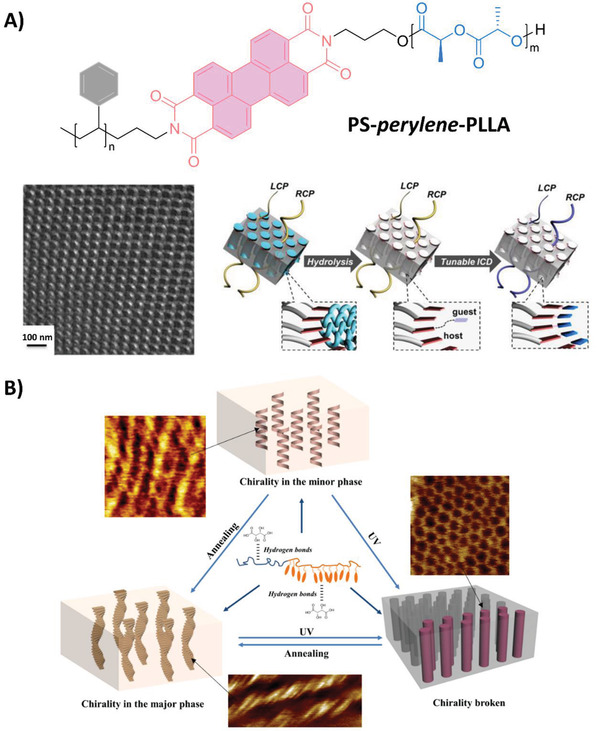
A) Chirality control and its chirality memory followed by subsequent chirality induction at the microphase‐separated interface of self‐assembled chiral PS‐*b*‐PL(D)LA. The chemical structures of PS‐perylene‐PLLA, TEM micrograph of the helical phase structures in PS‐perylene‐PLLA/PS‐PLLA blends and the schematic illustration of the control, memory, and induction process of chirality inside the phase structures. Adapted with permission.^[^ ^42^ ^]^ Copyright 2017, American Chemical Society. B) Illustration for the transfer, photoinduced breaking, and regeneration of the aggregation chirality in the film of an amphiphilic liquid‐crystalline BC doped with chiral tartaric acid. Here, the aggregation chirality was obtained by chiral transfer from the minor to the major phase through hydrogen bonds by optimizing the annealing condition along with the dopant amount. Reversible photo‐manipulation of the aggregation chirality was observed by the light‐induced phase transition of the photoresponsive azobenzene mesogens and the supramolecular cooperative motion. Adapted with permission.^[^ ^48^ ^]^ Copyright 2018, Wiley‐VCH.

However, the superiority of the chiral PLA‐based BCs enables them to function as reliable chiral templates because of the degradable characteristics of PLA. Ho et al. obtained the induced chirality of an achiral chromophoric joint of PLA‐containing chiral BCs. The achiral dyes adopted the preferential arrangement in a single handed helical array at the microphase‐separated interface. After the hydrolysis of the PLA block, this helical arrangement could be memorized, functioning as a chiral template for the further chirality induction of different achiral dyes, as shown in **Figure** **6**A.^[^ ^42^ ^]^ In addition, since the helical phase is an intermediate state in chiral PLA‐containing BCs, it would evolve into equilibrium double‐gyroid phases after annealing for an extended period.^[^ ^40^, ^43^ ^]^ The hydrolysis of the PLA block resulted in the formation of a nanoporous PS matrix with gyroid nanochannels, which could function as removable templates to fabricate nanoporous gyroid structures composed of all types of elements and materials for different functions. These functions include, nanoporous gyroid Pt for electrochemical catalysis, nanoporous gyroid metallic oxides for optoelectronic applications, nanoporous epoxy for protein adsorption, and nanoporous Ni/NiO/C composites for electrical properties.^[^ ^44^ ^]^

Chiral doping is also an effective approach for fabricating various helical phase structures for achiral polymers. Watkins et al. reported the formation of helical phases by directly doping a small chiral additive, tartaric acid (TA), into an achiral BC, poly(ethylene oxide)‐*b*‐poly(tert‐butyl acrylate) (PEO‐*b*‐PtBA). The handedness of the dopant determined the twist direction of the helical structures.^[^ ^45^ ^]^ Furthermore, Lu et al. fabricated double‐helical nanostructures by replacing the achiral BC with poly(1,4‐butadiene)‐*b*‐poly(ethylene oxide) (PBd‐*b*‐PEO) using such a doping method.^[^ ^46^ ^]^ One strength of this chiral doping strategy is that the dopant can be easily removed by solvents, while the asymmetric phase structures can be memorized after the removal of dopants. Based on this method, the chiral arrangement of gold nanoparticles with both single helical structures and double helical structures were successfully obtained.^[^ ^46^, ^47^ ^]^

Another advantage of the chiral doping method is that the interactions between dopants and specific blocks of BCs can be manipulated. Yu et al. obtained photoresponsive and phototunable helical structures by doping the enantiopure TA in a liquid‐crystalline BC (LCBC), poly(ethylene oxide) blocked with azobenzene‐containing poly(methylacrylate) (PEO‐*b*‐PM11AZ), where both blocks could form supramolecular interactions with the chiral dopants.^[^ ^48^ ^]^ As shown in Figure 6B, the controllable microphase separation of PEO‐*b*‐PM11AZ and the chirality transfer effect were elegantly combined. Through supramolecular interactions, chirality was transferred from the functional dopant to the aggregation, which directed the hierarchical self‐assembly in the composite system upon optimizing the annealing condition and the dopant fraction. The aggregation chirality disappeared upon photoirradiation with UV light because a photoinduced phase transition arose from the continuous phase of photoresponsive azobenzene mesogens, thereby resulting in the breakage of the helical aggregates along with the reorganization of the microphase‐separated nanostructures. Thus, the photo‐regulation of the supramolecularly self‐assembled helical morphologies in the condensed state was achieved in the LCBCs. This report introduces manipulatable functions into the simple, but effective, method for the construction of asymmetric phase nanostructures.^[^ ^48^ ^]^

## 4 Chiral Gels

The physical gel system is a typical soft matter with good stimulus‐responsive properties and extensive potential in numerous fields.^[^ ^49^ ^]^ Both the easily adjustable feature of solution systems and the self‐standing specialty of solid systems are combined in gels by bearing a certain amount of solvent in the solid skeleton.^[^ ^50^ ^]^ Moreover, the introduction of chirality increases the complexity of hierarchical structures and extends the functionality for chiral materials. In chiral gels, hydrogen bonding, hydrophobic interactions, *π*—*π* stacking, alkyl chain entanglement, and metallic coordination are the most common driving forces, which not only induce the self‐assembly of molecules, but can also transfer chirality. Since hydrogen bonding and hydrophobic interactions exist extensively in these systems as the assistant interactions for gel formation, we will mainly discuss the effect of *π*—*π* stacking, alkyl chain stacking and entanglement, and coordination interaction on the self‐assembly and chirality transfer behaviors in gel systems in this section. In fact, gels often form through multiple driving forces rather than single ones. Hence, we will comprehensively consider their contributions to chirality transfer and gel formation for the categorization in this section.

### 4.1 *π*—*π* Stacking Systems

Most *π*‐conjugated molecules tend to stack in a face‐to‐face manner in their aggregation states, and *π*—*π* stacking in chiral systems could function as the driving force for the formation of self‐assembled gelation. However, offset *π*—*π* stacking in systems exclusively consisting of achiral molecules could generate supramolecular chiral bias and break the symmetry of aggregates in gels. Liu et al. first accomplished the symmetry breaking in a supramolecular gel exclusively driven by *π*—*π* stacking of an achiral *C*_3_‐symmetric benzene‐1,3,5‐tricarboxylate substituted with methyl cinnamate in particular achiral organic solvents like cyclohexane.^[^ ^51a^ ^]^ The supramolecular chirality could not be controlled and assemblies with both handedness were observed, unless a small amount of chiral solvents was added to promote a predominant handedness. The chirality of the assemblies could be memorized after the removal of chiral solvents. Moreover, a strong tunable circularly polarized luminescence (CPLU) was observed in the supramolecular gels self‐assembled from a similar achiral *C*_3_‐symmetric molecule, where the three carboxylate linkers between the benzene core and substituted groups were replaced by amides (BTAC).^[^ ^51b^ ^]^ The CPLU intensity of these supramolecular gels was easily enhanced by mechanical stirring or doping chiral amines. In addition, the handedness of the CPLU signals could be controlled by the chirality of organic amines.

**Figure 7:**
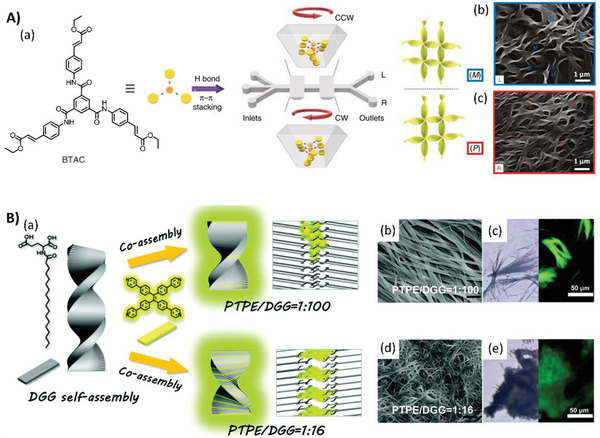
A) Microfluidic induced chiral self‐assembly of the BTAC building blocks in microvortices. (a) Molecular structure of achiral BTAC and the schematic illustration of the effect of the shear forces of enantiomorphic microvortices (CW and CCW rotating directions) on the mirror symmetry breaking process of achiral BTAC molecules. SEM images of the predominantly (b) M and (c) P twists for BTAC gels from the L‐ and R‐outlet, respectively. M and P chirality refers to left‐ and right‐handed helical twists, respectively. Adapted with permission.^[^ ^52^ ^]^ Copyright 2018, Springer Nature. B) Supramolecular chirality inversion based on the PTPE content. (a) Molecular structures of PTPE and gelator DGG. At a molar ratio of PTPE/DGG = 1:100, bunchy‐like nanofibers with significant left‐handed CPL signals are obtained. At PTPE/DGG = 1:16, the handedness of CPL is inverted to a mirrored right‐handed pattern. SEM images of PTPE/DGG co‐gels at the molar ratio of (b) 1:100 and (d) 1:16 (scale bar: 1 µm). Optical microscopy image (left side) and fluorescence microscopy image (right side) of co‐gels: (c) PTPE/DGG = 1:100 and (e) PTPE/DGG = 1:16. Adapted with permission.^[^ ^58^ ^]^ Copyright 2019, Royal Society of Chemistry.

Similar to the solution systems, the generation of supramolecular chirality in gels could be achieved using mechanical forces. The above‐mentioned achiral *C*_3_‐symmetric molecules with amide linkers could generate supramolecular chirality when self‐assembling in solutions or as gels through *π*—*π* stacking along with hydrogen bonding, induced by laminar chiral microvortices within asymmetric microchambers. The aggregation chirality in this case could be controlled by the rotational direction of the vortices.^[^ ^52^ ^]^ If the peripheral groups were transformed into carboxyl acid, which could produce stronger hydrogen bonds in the supramolecular gel system, near‐unity homochirality entities were also obtained; however, the direction of vortex mixing was unable to dictate the chirality (**Figure** **7**A).^[^ ^53^ ^]^ These studies indicate that a laminar chiral microflow can break supramolecular and aggregation symmetries, providing insight into the origin of natural homochirality of biological systems.

The aggregation of chiral molecules through *π*—*π* stacking usually form various chiral structures such as twist ribbons, helices, and nanotubes,^[^ ^10c^ ^]^ which could further transfer handedness to the nano‐assemblies of achiral guest molecules. Liu et al. reported the incorporation of chiral self‐assembly and aggregation‐induced emission (AIE) enabled by encapsulating guest AIE‐active achiral molecules in the nanotubes assembled from a *C*_3_‐symmetric chiral gelator.^[^ ^54^ ^]^ This approach assists in the fabrication of full‐color‐tunable CPLU by selecting suitable AIE compounds.

The supramolecular gelation process based on *π*—*π* stacking assisted by other interactions, such as hydrogen bonding and hydrophobic interactions, not only transfers the structural chirality information, but also transmits energy information. The chiral donor *π*‐gelator contained cyano‐substituted stilbene conjugated with *N,N′*‐bis(dodecyl)‐*L*(*d*)‐amine‐glutamic diamide. By co‐assembling this chiral donor *π*‐gelator with an achiral *π*‐acceptor, 9,10‐bis(phenylethynyl)anthracene, both chirality and energy transfer were simultaneously accomplished in one composite system.^[^ ^55^ ^]^ The co‐assembly process enabled the achiral acceptors to obtain circularly polarized energy and adopt a chiral alignment, thus exhibiting both supramolecular chirality and energy transfer amplified CPLU. However, in systems involving hydrogen bonding and *π*—*π* stacking, the controlled co‐assembly of a cyanostilbene‐appended glutamate compound (CG) with thioflavin T (ThT) and acridine orange could assist the chirality transferring from the chiral nanotubes formed by CG to the achiral acceptors. Upon exciting the donor CG or intermediate donor ThT, a donor–acceptor1–acceptor2 triad occurred, thereby resulting in a stepwise amplified CPLU.^[^ ^56^ ^]^ Efficient energy transfer could contribute to CPLU enhancement, thus leading to a higher luminescence dissymmetry factor. These reports demonstrate the cooperative effect of chirality and sequential energy transfer, which provides a thorough understanding of the natural light‐harvesting process occurring in chiral environments.

The stimulus‐responsive properties of some components in chiral gels exert a profound impact on the self‐assembled asymmetric structures, which will certainly influence the chirality and energy transfer. Liu et al. designed a photosensitive cinnamic acid derivative chiral gelator that could form dimers upon photoirradiation, and fabricated a chiroptically switchable helical self‐assembly system. Based on the *π*—*π* stacking and hydrogen bonding, superhelices could be obtained through the hierarchical chirality transfer, which would dramatically transform into nano‐kebabs due to the disturbance in the supramolecular interactions upon the photoirradiation of UV light. Such structural changes even caused the inversion of supramolecular chirality.^[^ ^57^ ^]^ However, nanoscale helicity could be conveyed to achiral fluorescent molecules by both structures, thus transferring energy information and inducing CPLU.

In addition, the stoichiometric ratio of different components can influence hierarchical chirality transfer and induce a chirality inversion behavior. Yin et al. prepared co‐gels with an achiral tetraphenylethylene derivative and chiral organic gelators of glutamic acid in chloroform. Through hydrogen bonding along with *π*—*π* stacking, chirality was transferred from the chiral gelators to the achiral *π*‐conjugated molecules with pyridine end groups, and then to the aggregate structures, thus inducing CPLU. However, both the handedness of the aggregates and the induced CPLU inverted after the stoichiometric ratio changed. This provides a simple strategy for regulating the handedness of CPLU in these supramolecular co‐gelation systems (Figure 7B).^[^ ^58^ ^]^

Achiral components sometimes play an important role in the asymmetric self‐assembly in co‐gelation systems. Liu et al. observed that purine nucleobases could trigger *N*‐(9‐fluorenylmethoxy‐carbonyl)‐protected glutamic acid to self‐assemble into hydrogels with helical structures, while pyrimidine nucleobases could not because the hydrogen bonding between guanine or adenine and glutamic acid enhanced *π*—*π* stacking and hydrophobic interactions in the co‐assembled systems, which also assisted the expression of aggregation chirality. Moreover, the helical nanostructures could transfer the chirality to an achiral fluorescence probe, thus inducing an enhanced CPLU.^[^ ^59^ ^]^ This paper might enable comprehension of the selective chiral assembly behaviors of biological systems.

### 4.2 Chain Stacking and Entanglement Assisted Gelation

The stacking and entanglement of chains usually occur in supramolecular systems containing a long flexible alkyl chain, thus providing a relatively strong supramolecular interaction for hierarchical aggregation and chirality transfer. In the gel systems enabled by the alkyl chain stacking and entanglement, *π*‐conjugated aromatic rings or functional groups that can form hydrogen and halogen bonds are not required, which releases the restriction in molecular structure design for gel systems. In addition to driving self‐assembly, the introduction of a long alky chain can increase the flexibility of assemblies, which is a desirable advantage for adaptive soft matter.

**Figure 8:**
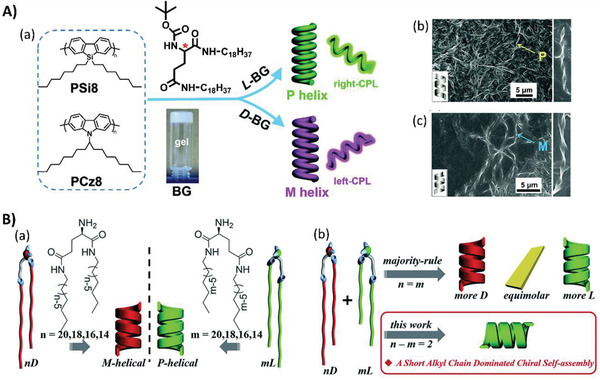
A) Co‐gelation and chiral transfer from the chiral gelator BG to the achiral main‐chain polymers PSi8 and PCz8. (a) Chemical structures of achiral PSi8 and PCz8, and the formation of P and M helices by doping L‐BG and D‐BG, respectively. Excited with 360 nm linearly polarized light, the co‐gels mirror‐handed circularly polarized luminescence. SEM images for the co‐gels of (b) PSi8/L‐BG with P helix and (c) PSi8/D‐BG with M helix. Adapted with permission.^[^ ^61^ ^]^ Copyright 2016, Royal Society of Chemistry. B) Self‐assembly of the chiral lipids that disobey the majority rule. (a) Chemical structures of enantiomerically pure D‐ and L‐lipids which form M‐ and P‐helices, respectively. (b) Schematic illustration of uncommon chirality transfer behaviors that do not follow the majority rule. Here, the mixing of two heterochiral lipids with mirror chiral head groups but a 2‐methylene discrepancy in the alkyl chain length leads to the homochiral composite nanotube, where the helical sense is determined by the molecular chirality of the lipid with the shorter alkyl chain regardless of their mixing ratios. Adapted with permission.^[^ ^62^ ^]^ Copyright 2019, Royal Society of Chemistry.

Multi‐responsive properties can be easily introduced into chiral gels by combining functional groups with gelator molecules containing a long alkyl chain. Liu et al. reported that the chiral sense in the organogel consisting of a spiropyran containing a long alkyl chain and a chiral cationic *L* (d)‐glutamate amphiphile (1‐(2‐(3‐(1,5‐bis(octadecylamino)‐1,5‐dioxopentan‐2‐yl)ureido)ethyl)pyridin‐1‐ium chloride, could be transferred to spiropyran chromophores via the entanglement of alkyl chains. Moreover, the chiroptical properties can be modified by photoirradiation or variations in the pH.^[^ ^60^ ^]^ The memorization of handedness could also be accomplished in the organogels containing a long alkyl chain. An amphiphilic glutamide based gelator was utilized to tune the achiral chromophores in main‐chain polymers into helical aggregates (**Figure** **8**A).^[^ ^61^ ^]^ The co‐gelation along with a supramolecular chirality transfer process was driven by the alkyl chain interactions between the polymer and gelator molecules. The aggregation helicity and the induced CPLU were memorized, even after the removal of chiral gelator molecules.

When the stacking and entanglement of alkyl chains function as the predominant driving force for gel formation and chirality transfer, the lengths of the alkyl chains significantly influence the self‐assembly. When two heterochiral lipids with mirror headgroups but a 2‐methylene discrepancy in the length of the alkyl chain were mixed, the majority rule for chiral transfer might be disobeyed (Figure 8B).^[^ ^62^ ^]^ Homochiral nanotubes were formed at aggregation level, but interestingly, their helicities were controlled by the molecular chirality of the lipids with shorter alkyl chains, regardless of the mixing ratio. The “induced conformation rearrangement” mechanism was used to explain such an abnormal phenomenon. Based on the interaction between the alkyl chains, the conformation of longer lipids required rearranging to match the shorter lipids, which helped to eliminate the orientation of the long lipids. Thus, the alkyl chain packing, hydrogen‐bonding of the amide groups in the lipids, and the orientation of the two lipids were perfectly matched, resulting in the molecular chirality of lipids with shorter alkyl chains dominating the chiral transfer process.

In complex co‐gel systems driven by chain entanglement, an induced‐fit mechanism could be applied to transfer chirality among hierarchical structures to generate complex chiral structures. Yashima et al. reported the formation of a “helix‐in‐helix” superstructure by encapsulating fullerene‐bound helical peptides into a helical syndiotactic poly(methyl methacrylate) (st‐PMMA) cavity.^[^ ^63^ ^]^ Induced by optically active alcohol or amine, st‐PMMA could fold into a preferred‐handed helical conformation and self‐assemble into organogels with an inner cavity. The helical cavity was used as the chiral host to endow achiral guests with optical activities, thereby resulting in asymmetric superstructures.^[^ ^64^ ^]^ Therefore, the helical conformation in the st‐PMMA backbone induced the same handedness in the encapsulated hexapeptide to form a crystalline st‐PMMA/peptide‐C_60_ inclusion complex with the helix‐in‐helix structures. These findings offer a rational design strategy for developing functional soft materials, which can serve as supramolecular nano‐reactors with the function of asymmetry catalysis.

### 4.3 Coordination Interaction Assisted Self‐Assembly

Coordination interaction is a special type of supramolecular interaction with properties extremely similar to those of covalent bonds. Coordination interactions usually exist between cations and electronegative atoms containing non‐bonded lone pair electrons. Due to the high binding energy and directionality of the coordination interaction, the stacking of molecules and supramolecules could be severely influenced, which should further affect their self‐assembly. For chiral gels, the introduction of coordination interactions could provide a really strong driven force for gel formation and supramolecular chirality transfer, resulting in stable hierarchical structures. Moreover, the high binding energy and directionality of coordination bonds makes the supramolecular complex thermally stable, which could function as a building block to fabricate various assembled structures at the multiscale level by further employment of other supramolecular interactions in the supramolecular system.

Among all the coordination interactions, the coordination between metal cations and heterocyclic compounds has been extensively studied because it offers a strategy to combine the unique properties of metallic compounds and organic systems together. Stang et al. prepared alanine‐based chiral organoplatinum(II) metallacycles rhomboids 1^d^/1^l^ and hexagons 2^d^/2^l^, using the Pt(II)←pyridyl directional bonding approach.^[^ ^65^ ^]^ The subsequent self‐assembly was driven by hydrogen bonding, hydrophobic, and *π*—*π* interactions among the metallacycles. As a result, the concentration played an important role in the assembly process. At a low concentration, where the other aforementioned supramolecular interactions were relatively weak, the metallacycles tended to assemble into nanospheres, while the chiral metallogels were generated at a high concentration under the cooperation of hydrogen bonding, hydrophobic and *π*—*π* interactions. It was also demonstrated that the molecular chirality of the metallacycles was transferred via the self‐assembly process and resulted in the formation of helices at the supramolecular level, leading to the formation of chiral gels consisting of microscopic chiral nanofibers with well‐defined helicity.

**Figure 9:**
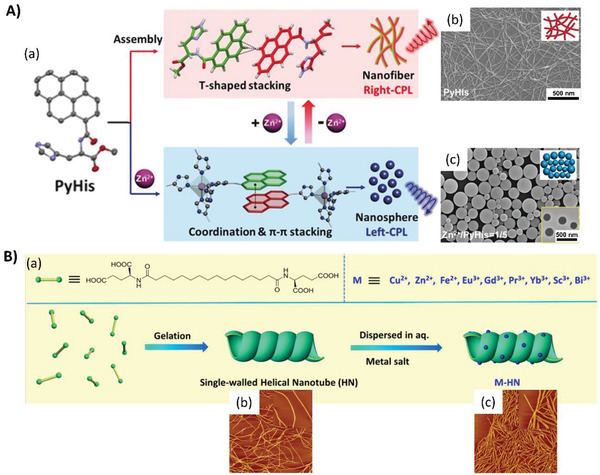
A) Inversion and switching of the nano‐assemblies and their CPL regulated by the combination of coordination and *π*‐stacking. (a) Schematic illustration. (b) SEM image of PyHis xero‐gel. (c) SEM image of PyHis in the presence of Zn^2+^ (Zn^2+^/PyHis = 1/5), and the inserted TEM image at the bottom‐right corner. Adapted with permission.^[^ ^66^ ^]^ Copyright 2019, Wiley‐VCH. B) Formation of the metal‐helical nanotube (M‐HN). (a) Chemical structures of the chiral ligand and the schematic illustration of the formation of the metal‐helical nanotube. (b) AFM images of the self‐assembled helical single‐walled nanotube from the ligand. (c) AFM images of Bi(III)‐HN after loading 1/50 mol of Bi^3+^. The size of images was 5 × 5 µm^2^; the size of the enlarged image in parts (b) and (c) is 1 × 1 µm^2^. Adapted with permission.^[^ ^67^ ^]^ Copyright 2016, American Chemical Society.

The self‐assembly pathways can be switched by coordination interactions. Liu et al. reported the modulation of the self‐assembly pathways through a cooperative Zn^2+^ coordination and *π*‐stacking among the pyrenes. Thus, pure pyrene‐conjugated histidine was stacked in an unusual T‐shaped *π*‐stacking to form a nanofiber structure, exhibiting P‐chirality and right‐handed CPLU. However, the chromophore packing model was altered from T‐shaped to *π*‐*π* stacking such that the nanofibers transformed into nanospheres and inversed into M‐chirality showing left‐handed CPLU upon Zn^2+^ coordination, as shown in **Figure** **9**A.^[^ ^66^ ^]^ In general, the opposite supramolecular chirality as well as CPLU signals could be obtained by the cooperation of coordination interactions with *π*—*π* stacking. This paper presents an elegant strategy for the preparation of manipulatable functional chiral materials by switching the self‐assembly pathways.

The helical metal‐coordinated superstructures can be used as catalysts for asymmetric reactions. Recently, Liu et al. reported that complex chiral gels formed upon the coordination of a metal ion with a single‐walled nanotube self‐assembled from an l‐glutamic acid terminated bolaamphiphile in water.^[^ ^67^ ^]^ The utility of different metal ions could help create various metal‐helical nanotubes, as shown in Figure 9B, where the coordination points could function as efficient catalyst sites for two model asymmetric reactions. For example, the metal ion Bi(III) coordinated helical nanotubes could catalyze the asymmetric Mukaiyama aldol reaction with a high enantioselectivity (up to 97% ee) in an aqueous system. Another metal ion, Cu (II) coordinated helical nanotubes, could catalyze the asymmetric Diels–Alder reaction with up to 91% ee within 60 min. This paper adequately presents the advantages of metal‐coordinated chiral gel systems in the application of nanocatalysts, and delivers an approach to asymmetric synthesis with a new strategy by the employment of hierarchically self‐assembled chiral nanostructures.

### 4.4 Weak Interactions between Organic and Inorganic Compounds

Based on the weak interactions in organic‐inorganic composite systems, co‐assembly along with chirality transfer and energy transfer can be achieved. Liu et al. prepared organic‐inorganic plural gels from lanthanide‐doped inorganic upconverting nanoparticles using chiral nanotube encapsulation.^[^ ^68^ ^]^ The organic component would self‐assemble into chiral nanotubes driven by hydrogen bonding and hydrophobic interactions. The handedness of the organic assembled nanotubes was transferred to the encapsulated upconverting nanoparticles, leading to a chiral arrangement of the doped nanoparticles, which further induced CPLU at a wide range of wavelengths from UV to near infrared light. In addition, they found that the UV section of the upconverting CPLU could be used to initiate the enantioselective polymerization of diacetylene, endowing such organic‐inorganic plural gel systems with good application potentials. This report inspires a novel approach toward functional CPLU‐active materials with a highly efficient and large dissymmetry factor, which may help push forward the application of the chiral optical materials.

Achiral perovskite nanocrystals could also co‐assemble with chiral lipid gelators, demonstrating an induced CPLU through the supramolecular chirality transfer.^[^ ^69^ ^]^ The doped nanocrystals might follow the chirality of the gel structure formed by the chiral lipid to produce a chiral packing. By doping various colorful achiral perovskite nanocrystals, the co‐assembled chiral gels could exhibit colorful circularly polarized emission, and thus enabled CPLU to show a variety of colors, which could be further applied to facilitate flexible CPLU devices.

## 5 Asymmetrical Self‐Assembly at the Interface

Apart from solutions, condensed states, and gelation systems, there is another special environment for materials that can profoundly impact the supramolecular chirality transfer during the hierarchical self‐assembly, the interface. Generally, the most common interfaces for self‐assembled systems are gas–liquid and liquid–solid interfaces. Under the restriction of interface interactions such as interfacial tension and different affinities in the adjacent phases, molecular motion can be limited at the 2D interface. Thus, molecules can self‐organize into ultrathin structures like mono‐ or bi‐molecular layer structures, which are fundamentally different from the self‐assembled structures in solutions, condensed states or gels and exhibit some unique properties, for instance, the Langmuir–Blodgett (LB) films.^[^ ^70^ ^]^ The introduction of chirality will also induce asymmetric supramolecular structures, but the stacking and arrangement of molecules should be distinguished from that in the other aforementioned systems due to the confined self‐assembly process at the interface.

**Figure 10:**
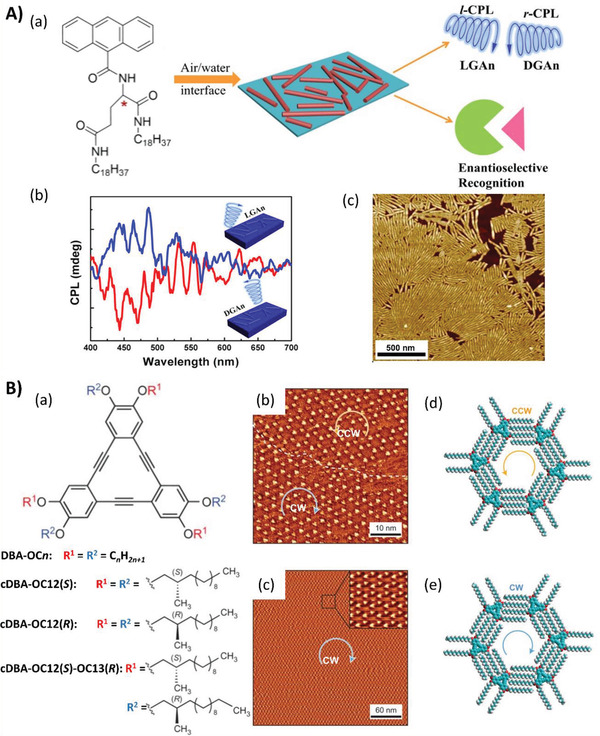
A) Chiral self‐assembly in LB film. (a) Chemical structures of enantiopure *N,N*′‐bis(octadecyl)‐L(D)(anthracene‐9‐carboxamido)‐glutamic diamide (abbreviated LGAn or DGAn), and schematic illustration of the formation of LGAn or DGAn nanorod films at the air/water interface. The films show CPL depending on the handedness of the formed nanorods. (b) CPL spectra of DGAn and LGAn nanorods films (c) AFM images of one‐layer LB films of LGAn deposited onto a freshly cleaved mica surface from the water subphase. Adapted with permission.^[^ ^71^ ^]^ Copyright 2019, American Chemical Society. B) Dynamic control over supramolecular chirality at the solution–solid interface. (a) Molecular structures of chiral and achiral DBAs. STM images of the DBA monolayer formed at the selected interface before (b) and after (c) thermal annealing. Molecular arrangement of (d) counterclockwise (CCW) and (e) clockwise (CW). Adapted with permission.^[^ ^72^ ^]^ Copyright 2016, Springer Nature.

The LB method is very efficient for preparing ultrathin nanofilms at the gas–liquid interface. For instance, nanorod structured monolayers were fabricated at the air/water interface using an LB technique with the self‐assembly of two enantiomeric gelator molecules containing an anthracene moiety.^[^ ^71^ ^]^ During this process, chiral sense was transferred to the assemblies, enabling the LB films to demonstrate both optical activity and CPLU properties, as shown in **Figure** **10**A. It was also found that the dissymmetric factors of the CPLU in the LB films were enhanced by nearly five times those in gel systems because of the more compact 2D stacking mode of molecules in LB films. In addition, the organized nanofilms demonstrated enantioselectivity to chiral species, whereas the molecular solution did not.

The self‐assembly of molecules at the liquid–solid interface is another interesting topic where the “sergeant and soldiers” principle is also applicable. Recently, Feyter et al. combined the “sergeant and soldiers” principle with temperature‐dependent molecular self‐assembly to unravel a particular chiral amplification mechanism at the solution–solid interface (Figure 10B). They described the fabrication of a homochirality surface where the majority handedness of the sergeant‐soldiers system was amplified or entirely reversed depending on the concentration of solution after thermal annealing.^[^ ^72^ ^]^ It was deduced that the annealing treatment enlarged the chiral bias of the system, resulting in the chirality amplification or reversion phenomena of the initial majority handedness during the supramolecular chirality transfer process. These results provide a novel enantioselective host–guest interaction‐based amplification pathway to induce the generation of opposite handedness if the concentration of the building blocks is high enough. Moreover, the fabrication of controllable and manipulatable homochirality supramolecular interfaces with either handedness can be achieved by introducing a single type of chiral modifier. This report also provides a detailed understanding of the roles that subtle intermolecular and interfacial interactions play in supramolecular chirality transfer and the induced asymmetric hierarchical self‐assembly.

## 6 Summary and Outlook

Herein, we discussed recent progresses in supramolecular chirality transfer leading to asymmetric hierarchical self‐assembly under different environments. In solutions, the inherent properties of the solvents such as solubility, polarity, and chirality significantly influence the molecular dynamics and the expression of chirality, thus resulting in various micro‐assemblies. Due to the unrestricted molecular dynamics in solution systems, it is facile to manipulate the supramolecular structures by adjusting the interactions among the solute molecules, between the solutes and the solvents, or by the application of external fields. However, their stability requires improvement by post processing methods such as crosslinking. There is a completely different situation in the condensed state, where the compact molecular stacking and the absence of solvents restrict the mobility of molecules, providing excellent stability of the internal superstructures but making them difficult to be controlled and modulated. Thus, the design of molecules and the optimization of treatment conditions become particularly important in condensed state systems. Annealing treatments including thermal annealing and solvent annealing are the most effective approaches to facilitate molecular motions in condensed states, leading to self‐assembly behaviors like crystallization, reorganization, and ordered phase separation. Hence, the introduction of chirality creates a bias that can be transferred and amplified, making the expression of the asymmetric sense in various chiral assemblies, crystalline phases, and phase structures at the nanoscale level. Chiral gel systems possess the advantages of both the solution and the condensed state because of their spatial structures that bear a certain amount of the solvent in the 3D skeleton. As a result, the easily adjustable property of solution systems and self‐standing characteristics of solid systems can be incorporated in chiral organogels, endowing them with multi‐functions as intelligent chiral materials. Supramolecular interactions including hydrogen bonding, hydrophobic interactions, *π*—*π* stacking, alkyl chain entanglement, and metallic coordination play very significant roles in the formation of gels with 3D spatial structures, the hierarchical self‐assembly process, and the transfer, amplification, and memorization of chirality in such gelation systems. The interfaces provide a unique environment that can profoundly impact the supramolecular chirality transfer during hierarchical self‐assembly. The interface effect restricts molecular motion and their arrangement at the 2D interface plane, thus resulting in distinguished confined self‐assembly behaviors to form ultrathin mono‐ or bi‐molecular layers. The chiral superstructures at the interface assist the realization of functional nano‐films or homochiral surfaces which could exhibit enantioselectivity toward chiral species.

In addition, supramolecular interactions, which are the major driving forces for the ordered organization and arrangement of molecules, also significantly influence the expression, transfer, and amplification of the chiral sense and the hierarchical asymmetry at multiscale levels. In general, van der Waals forces, hydrogen bonding, hydrophobic interactions, coordination interactions, electrostatic interactions, *π*—*π* stacking, and the entanglement of alkyl chains are the most common intermolecular interactions that can lead to hierarchically self‐assembled structures. As a result, a broad diversity of nanoarchitectures can be fabricated based on the inherent properties of various supramolecular interactions such as their strength and directionality. There are several methods to introduce chiral bias into supramolecular systems by drawing in molecules with inherent chiral centers or chiral axis, breaking supramolecular symmetry through controlling the self‐assembly process, and employing asymmetric external fields.

There has been considerable development in the transfer and amplification of supramolecular chirality at multiscale levels along with the potential applications. However, the emergence of chirality and method to control the hierarchically chiral structures in complex systems like bio‐systems has not been properly elucidated. Hence, the exploration of the origin of symmetry breaking at different levels, the relationships among chiral structures at different length scales, and the factors that influence the expression of chirality at hierarchical levels such as the self‐assembly process and the environmental aspects, are essential for comprehending the emergence and functions of chirality in nature as well as directing the design strategies for chiral materials.

The above strategies to create hierarchical chiral structures through self‐assembly and chirality transfer are typical bottom‐up methods, which can lead to complicated nanostructures including single, double, and dendritic helices as well as other twisted structures demonstrating optical activity. In addition, top‐down methods have been utilized to fabricate macroscopic chiral structures. By preparing multi‐layered films or fibers with distinct thermal and mechanical properties of each layer, suitable treatment such as thermal annealing and photo‐irradiation with a programmable shaping procedure, can activate their interior asymmetry and help create helical macrostructures, as reported by Ikeda^[^ ^73^ ^]^ et al., Zhao^[^ ^74^ ^]^ et al., and Yu^[^ ^75^ ^]^ et al. These papers indicate that hierarchical chirality can also be generated by top‐down methods, which might be extrapolated to nanoscale levels and incorporated into supramolecularly self‐assembled systems to create a chiral bias with the help of the distinct respective thermal or mechanical characteristics of different components.

The chiral nanostructures based on self‐assembly and hierarchical chirality transfer can be used as nanotemplates for the preparation of inorganic or metallic functional materials with chiral microstructures.^[^ ^44^, ^46^, ^47^, ^48^, ^76^ ^]^ The transfer and amplification of chirality from molecular scale to aggregation level can enhance the chiral properties, providing a stereo‐geometrical environment or chiral space and endowing them with special functions such as chiral sensing, chiral separation, asymmetry catalysis, and several bio‐effects. Moreover, photonic and plasmonic properties with chiral features can also be achieved by fabricating the chiral arrangement of inorganic or metal nanoparticles based on supramolecular chirality transfer and hierarchical self‐assembly, or combined with novel top‐down approaches when necessary.

## Conflict of Interest

The authors declare no conflict of interest.
